# The high methylation level of a novel 151-bp CpG island in the ESR1 gene promoter is associated with a poor breast cancer prognosis

**DOI:** 10.1186/s12935-021-02343-7

**Published:** 2021-12-04

**Authors:** Laura Itzel Quintas-Granados, Hernán Cortés, Manuel González-Del Carmen, Gerardo Leyva-Gómez, Lilia Patricia Bustamante-Montes, Miguel Rodríguez-Morales, Edgar Yebran Villegas-Vazquez, Israel López-Reyes, Sofía Lizeth Alcaraz-Estrada, Jorge Sandoval-Basilio, Ernesto Soto-Reyes, Javad Sharifi-Rad, Gabriela Figueroa-González, Octavio Daniel Reyes-Hernández

**Affiliations:** 1 Universidad Mexiquense del Bicentenario, Unidad de Estudios Superiores Tultitlán, Mexico, Mexico; 2 Laboratorio de Medicina Genómica, Departamento de Genómica, Instituto Nacional de Rehabilitación Luis Guillermo Ibarra Ibarra, 14389 Mexico City, Mexico; 3 Facultad de Medicina, Universidad Veracruzana, Ciudad Mendoza, 94740 Veracruz, Mexico; 4 Departamento de Farmacia, Facultad de Química, Universidad Nacional Autónoma de México, 04510 Mexico City, Mexico; 5 Decanatura Ciencias de la Salud, Universidad Autónoma de Guadalajara, 44670 Guadalajara, Jalisco México; 6 Laboratorio de Genómica, Instituto Nacional de Cancerología, 14080 Mecixo City, México; 7 Departamento de Biotecnología y Bioingeniería del Centro de Investigación y de Estudios Avanzados del IPN, Mexico City, Mexico; 8 Colegio de Ciencias y Humanidades, Plantel Cuautepec, Universidad Autónoma de la Ciudad de México, Mexico City, Mexico; 9 División de Medicina Genomica, Centro Médico Nacional “20 de Noviembre”-ISSSTE, Mexico, 03100 Mexico City, Mexico; 10 Laboratorio de Biología Molecular, Universidad Hipócrates, Acapulco, Gro. México; 11 Laboratorio de Investigación Clínica, Facultad de Medicina, Universidad Autónoma de Guerrero, Acapulco, Gro. México; 12 Departamento de Ciencias Naturales, Universidad Autónoma Metropolitana-Cuajimalpa (UAM-C), Mexico City, Mexico; 13 Facultad de Medicina, Universidad del Azuay, Cuenca, Ecuador; 14 Laboratorio de Farmacogenética, UMIEZ, Facultad de Estudios Superiores Zaragoza, Universidad Nacional Autónoma de México, 09230 Mexico City, México; 15 Laboratorio de Biología Molecular del Cáncer, UMIEZ, Facultad de Estudios Superiores Zaragoza, Universidad Nacional Autónoma de México, 09230 Mexico City, México

**Keywords:** Breast cancer, ESR1 methylation, Mexican women, Menopausal stage

## Abstract

**Background:**

The *ESR*1 gene suffers methylation changes in many types of cancers, including breast cancer (BC), the most frequently diagnosed cancer in women that is also present in men. Methylation at promoter A of *ESR*1 is the worse prognosis in terms of overall survival; thus, the early detection, prognostic, and prediction of therapy involve some methylation biomarkers.

**Methods:**

Therefore, our study aimed to examine the methylation levels at the *ESR*1 gene in samples from Mexican BC patients and its possible association with menopausal status.

**Results:**

We identified a novel 151-bp CpG island in the promoter A of the *ESR*1 gene. Interestingly, methylation levels at this CpG island in positive ERα tumors were approximately 50% less than negative ERα or control samples. Furthermore, methylation levels at *ESR*1 were associated with menopausal status. In postmenopausal patients, the methylation levels were 1.5-fold higher than in premenopausal patients. Finally, according to tumor malignancy, triple-negative cancer subtypes had higher *ESR*1 methylation levels than luminal/HER2+ or luminal A subtypes.

**Conclusions:**

Our findings suggest that methylation at this novel CpG island might be a promising prognosis marker

**Supplementary Information:**

The online version contains supplementary material available at 10.1186/s12935-021-02343-7.

## Introduction

One of the most common cancers among women is breast cancer (BC), the second leading cause of cancer mortality in women [1]. Although this cancer occurs in men and women, statistics show that only 1 of every 100 cases of BC is diagnosed in men. In premenopausal women (younger than 50 years), BC is more frequent in low-income and middle-income countries than in high-income countries. In contrast, BC in postmenopausal women (50 years and older) is more common in higher-income countries [2].

Different risk factors for BC have been identified in premenopausal and postmenopausal women. For instance, excessive body weight and abdominal adiposity are important risk factors in postmenopausal ages [3]. On the other hand, risk factors for premenopausal BC are mainly reproductive. For instance, having few or no children or having children later in life reduces the risk of triple-negative BC (TNBC) but increases the risk of hormone-receptor-positive tumors [4]. Remarkably, BC therapeutic management and prognosis are different in premenopausal and postmenopausal patients. For example, the breast density in premenopausal women hinders early detection of BC. Furthermore, the BC subtype in young and older patients has important implications in survival and prognosis. In this regard, estrogen receptor-positive (ER+) tumors have a better prognosis in postmenopausal ages [2].

Epigenetic regulation in cancer, such as DNA methylation of cytosine-guanine dinucleotides (CpG), histone post-translational modifications, alterations in non-coding RNA expression, and recruitment of chromatin modifications have crucial roles in cancer initiation and progression [5]. In BC, hypermethylation at specific gene sites, mainly at CpG islands, is involved in oncogenes expression [6, 7]. Hypermethylation in BC includes genes encoding for BRCA1 protein [8], TIMP metallopeptidase inhibitor 3 (TIMP3) [9], Ras association domain family 1 isoform A (RASSF1A) [10], retinoic acid receptor β (RARβ) [11], and estrogen receptor-alpha (ERα) [12], among others. DNA methyltransferases 1 (DNMT1) and 3A (DNMT3A) levels are associated with hypermethylation of the *ESR1* gene and decreased expression of ERα, its encoded protein [12].

Molecules expressed in BC, such as estrogen receptors (ER), progesterone receptors (PR), and human epidermal growth factor receptor 2 (HER2), help to categorize the tumors into five subtypes such as luminal A and B, HER2 enriched, TNBC, or basal-like, and normal-like BC [13]. ER-negative cases seem to be more aggressive and confer a worse prognosis than ER-positive [14]. Thus, ERα is considered a prognostic biomarker suitable for predicting endocrine therapy response because 60% of ER-positive and 8% of ER-negative tumors showed an objective response. *ESR*1 methylation was observed in 80% of TNBC, 60% of HER2-enriched, 28% of luminal A, and 36% of luminal B cases [15]. A worse prognosis in overall survival correlated with methylation of the *ESR*1 promoter [16]. Likewise, metastatic tumors contained lower levels of ERα compared to primary tumors [17]. Moreover, 40–85% of BC patients presented hypermethylation of the *ESR*1 gene [18] that highly correlated with ER-negative/progesterone receptor (PR) negative conditions, suggesting that *ESR*1 methylation status strongly contributes to tumor phenotypes [19]. On the other hand, in free circulating DNA samples, the *ESR*1 methylation profile is correlated with ER-negative status and may be associated with resistance to hormonal treatment in BC patients [15]. Besides, the lack of response to exemestane treatment in BC patients related to the hypermethylation of *ESR*1 [20] and ERα's in vitro reactivation reveals sensitivity to tamoxifen in hypermethylated *ESR*1 samples.

Located in chromosome 6 (chr6), the *ESR1* gene contains approximately 475,472 bases, including the 140 kb section containing the eight protein-coding exons and eight regulatory elements utilized in a tissue-specific manner [21]. ER-positive BC primarily operates the proximal promoter (A promoter), whereas promoters B and C reported less activity [22–25]. Overlapping in the A promoter is more frequent than C promoter's overlapping in ER-positive primary patient samples [23].

Since endocrine therapy resistance in patients involves an ER dysregulated expression, the investigation about *ESR*1 will allow a better knowledge of the treatment effectiveness for BC. Since genetic modifications might contribute to the incidence of BC, this study aimed to investigate the methylation levels at the *ESR*1 gene in samples from Mexican BC patients and correlate these findings with the menopausal status. We found that the proximal promoter of the *ESR*1 gene contains a 151-bp CpG island located between the transcription start site (+ 1) and the ATG codon. Moreover, our findings indicated that methylation levels at 151-bp CpG island of the *ESR*1 gene are associated with menopausal status. Finally, according to tumor malignancy, TNBC subtypes had higher methylation levels than luminal/HER2+ or luminal A types, indicating that methylation at this novel CpG island might be a prognosis marker.

## Materials and methods

### Study population and ethics statement

This study enrolled a cohort of 20 women controls and 38 women with BC diagnosis between 2018 and 2019. All women who participated in this research had at least two generations born and lived in Mexico.

Participant women attended the Hospital Juárez de México (Mexico City, Mexico) for a breast biopsy because they felt a lump when performing a breast self-examination. The biopsies were analyzed immunohistochemically, and samples diagnosed with hyperplasia were considered as controls. All patients included in this research received no treatment at the time of sampling. Participants were included in this protocol after signing a written informed consent. We subdivided BC samples into ER+ or negative (ER−) groups, resulting in 19 patients per group. The immunohistochemical analysis performed by the Oncology and Pathology Services at the Hospital Juárez de México supported the categorization. The medical records endorsed the clinicopathological characteristics, such as menopausal state, cancer family history, and age at diagnosis time. All procedures performed in this research were performed according to the Code of Ethics of the Helsinki Declaration. The Ethics Committee from the Hospital Juárez de México approved this study (HMJ 2231/13-B).

### Nucleic acids isolation from tumor samples

Breast biopsies were stored at − 70 °C until their analysis. For DNA extraction, 200 mg of tissue was disrupted with mortar in the presence of liquid nitrogen. The frozen powder was transferred to a 1.5 ml tube, and 750 μl of lysis buffer (50 mM Tris–HCL, pH8.0; 25 mM EDTA, 400 mM NaCl, 10% SDS and, 10 mg/mL Proteinase K) was added and incubated for 1 h at 60 °C with agitation. Then, RNase (3 μl) was added to each sample, mixed carefully, and set at 37 °C for 30 min. Samples were kept at room temperature for 5 min, and then 200 μl of protein precipitation solution (6 M NaCl, 8 M guanidine hydrochloride (pH 8.0), and 0.49 M potassium acetate solution) were added. Samples were vortexed vigorously and kept on ice for 5 min. Samples were centrifugated at 16,000×*g* for 4 min to pellet debris and proteins. Supernatants were separated and collected in a clean tube. Then, 600 μl of 100% isopropyl alcohol was added and mixed carefully. Samples were centrifugated as described above. DNA pellet was washed three times with 70% ethanol. Finally, ethanol residues were eliminated, 100 μl of resuspension buffer (10 mM Tris–HCl, pH 8.0; 1 mM EDTA) was added, and DNA samples were kept at − 20 °C until further analysis.

RNA isolation from tissues was performed using the TRIzol Reagent (Invitrogen). Briefly, 20 mg of the biopsy was disrupted with mortar in the presence of liquid nitrogen. The frozen powder was transferred to a 1.5 ml tube, mixed with 1 ml of TRIzol reagent, and incubated at − 70 °C for 20 h. Then, samples were kept on ice until defrosting, and 200 μl of samples were transferred to a new tube and gently mixed with 40 μl chloroform. Samples were chilled ice for 4 min and centrifugated at 11,000 rpm for 12 min at 4 °C. The aqueous layer was transferred to a 1.5 ml tube, mixed with 500 μl isopropyl alcohol, and incubated at − 70 °C for 24 h. Samples were kept on ice until defrosting, centrifugated at 11,000 rpm for 5 min, and the pellet was washed with 200 μl ethanol and centrifugated at the same conditions. Pellet was resuspended in RNase-free water. DNA and RNA quantity and quality were determined using a spectrophotometer and gel electrophoresis, respectively.

### Analysis of* ESR1* methylation

Bisulfite modification of genomic DNA was performed using the MethylCode Bisulfite Conversion Kit (Invitrogen), and methylation in *ESR*1 was measured from BC patients' biopsies. Briefly, 500 ng of genomic DNA were denatured by incubation with 130 μl of CT Conversion Reagent for 10 min at 98 °C, followed by 150 min at 65 °C, and finally, samples were kept at 4 °C for 20 h. Modified DNA was purified using a spin column following the manufacturer's instructions and eluted with 10 μl of elution buffer. Samples were stored at − 20 °C until their use.

Primer sequences for the *ESR*1 gene for methylated sequences (M) were the following: forward primer 5′-TGCACTTGCTCCCGTCGGGTC-3′ and reverse primer 5′-AACCGGCGGGCCACCTGGAA-3′. The primer sequences for the *ESR*1 unmethylated sequences (U) were the following: forward primer 5′-GATTGTATTTGTTTTTGTTGGGTT-3′ and reverse primer 5′-AACCAACAAACCACCTAAAAAAA-3′. The cycling conditions were as follows: initial denaturation at 94 °C for 5 min, followed by 40 cycles at 94 °C for 30 s, at 58 °C for 45 s, and 72 °C for 60 s, the final extension was at 72 °C for 8 min.

Genomic DNA isolation allows the obtention of positive and negative methylation controls from healthy women's whole blood. The positive control was artificially methylated. Briefly, genomic DNAs (1 µg) were mixed with 2 µl of 10× NEBuffer2, 2 µl of SAM, and 1 µl of methyltransferase M.SssI enzyme. Samples were incubated 60 min at 37 °C, followed by incubation at 65 °C for 20 min to stop the reaction. Then bisulfite modification was performed as mentioned above. For negative controls, genomic DNA was subjected to bisulfite conversion without pretreatment. Finally, bisulfite sequencing PCR reactions were analyzed by densitometry, and normalized data were used to create all graphs.

### *ESR1* expression analysis

For cDNA synthesis, 2 µg of total RNA were reverse-transcribed using the Superscript II (Invitrogen) and the oligo-dT primer (500 µg/ml) according to instructions of the manufacturer. PCRs were performed using an ABI-PRISM 7000 Sequence Detector System (Applied Biosystems, Branchburg, NJ). Measurements of the relative amount of *ESR*1 in tumor samples were conducted in a single PCR reaction to normalize the number of target copies to that of the *18S* rRNA gene using the critical threshold cycle (*Ct*). Reaction mixture consisted of cDNA (2 ml), 1× TaqMan Universal Master Mix (Applied Biosystems, USA) and 0.9 mM primers and 0.25 mM of TaqMan probes (Thermo Scientific) for *ESR*1 (Hs00174860_ml) and 18S-ribosomal RNA (Hs99999901_sl). We used the conditions indicated by the manufacturer for the RT-PCR reactions.

### Statistical analysis

Pathological characteristics were summarized through descriptive analysis. Categorical variables were described through frequency distribution, whereas continuous variables were reported through the median and standard deviation (SD). Associations between promoter methylation, pathological characteristics, and transcriptional expression level were explored through χ^2^ or ANOVA tests by Prism software (GraphPad, San Diego, CA, USA).

## Results

The study population comprised 38 BC patients and 20 controls. For premenopausal women, the mean of age in cases was 41.33 ± 5.97, whereas in controls was 34.80 ± 12.42. For postmenopausal women, the mean of age in cases was 57.58 ± 12.56 and in controls was 49.60 ± 20.21. Table 1 represents the descriptive statistics of variables in the groups. According to the cancer family history, cases and controls were statistically different (*P* = 0.0410). Furthermore, we observed a difference in the premenopausal stage between BC and controls (*P* = 0.0016). When we analyzed the immunohistochemical profile, we found a statistically significant difference in the expression of HER2+ (*P* = 0.0457) (Table 1).

**Table 1:** Clinicopathological characteristics of the studied population

	Case	Control	*P*
	*n* (%)	*n* (%)	
Cancer family history			
Yes	24 (63.16)	7 (40.0)	0.0410*
No	14 (36.84)	13 (60.0)	

	*n* (%), age^+^	*n* (%), age^+^	
Menopause			
Premenopause	12 (31.6) 41.33 ± 5.97	15 (75.0) 34.80 ± 12.42	0.0016*
Postmenopause	26 (68.4) 57.58 ± 12.56	5 (25.0) 49.60 ± 20.21	
Cancer stage grouping			
I	1 (2.6)	NA	
II	18 (47.4)	NA	
III	15 (39.5)	NA	
IV	4 (10.5)	NA	
Phenotype			
Luminal/HER2+	11 (28.9)	NA	
HER2+	11 (28.9)	NA	
Luminal A	4 (10.5)	NA	
Mixed phenotype^a^	7 (18.4)	NA	
Triple-negative	5 (13.2)	NA	

	Case, *n* (%)	Control, *n* (%)	*P*
Immunohistochemical profile^b^			
Estrogen receptor (ER)	Positive	Negative	
Triple-negative	19 (50.0)	19 (50.0)	NA
Progesterone receptor	18 (47.4)	20 (52.6)	NA
HER2+	26 (68.4)	12 (31.6)	NA

*NA* not applicable, *n* sample size

*P* = statistically significant value was considered as *P* < 0.05 using Chi-square test

^a^Population expressing both: and estrogen receptor or progesterone receptor

^b^Comparative analysis into case group only

**Fig. 1:**
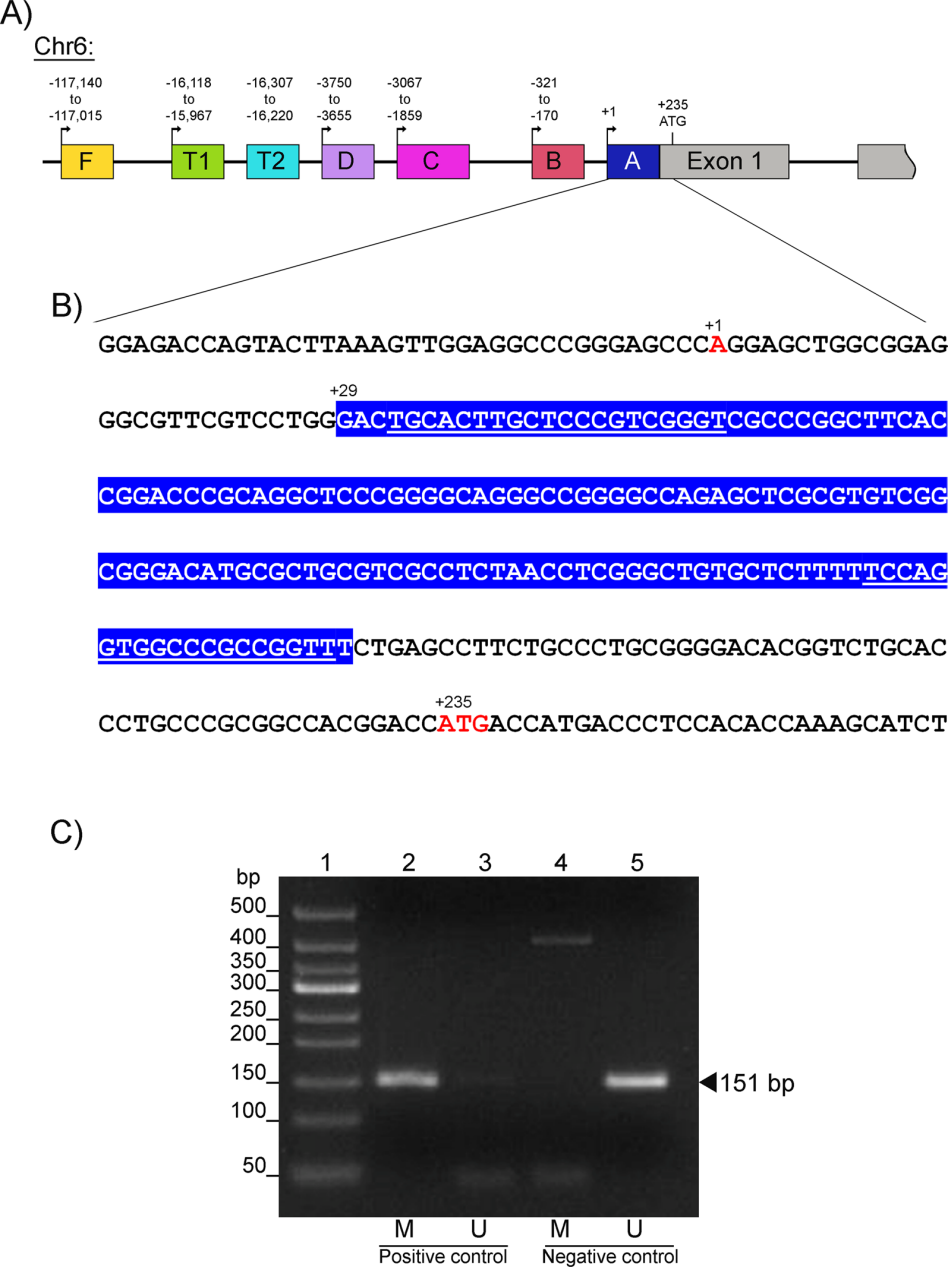
The sequence of the CpG island in *the ESR1* gene. **A** *ESR*1 promoter region scheme in chromosome 6 (Chr6). The 300-kilobases *ESR*1 gene contains seven promoters (color boxes) utilized in a tissue-specific manner and eight exons (only exon 1 is shown). **B** Assessed DNA sequence in the proximal promoter. A 151-bp sequence (blue box) located in the A promoter between the transcription start site (+ 1) and the ATG codon (+ 235) was evaluated. Underlined sequences corresponded to the primers used for methylation analysis. Nomenclature and numbering are based on previous reports [21, 54]. **C** Methylation-specific PCR analysis of *ESR*1. Electrophoretic profile of methylated (M) (lanes 2 and 4) and unmethylated (U) (lanes 3 and 5) samples from positive (lanes 2 and 3) and negative (lanes 4 and 5) controls using genomic DNA from patients with fibroadenoma. 50-bp DNA Ladder (lane 1). Arrowhead indicates the amplicon size. Genomic coordinates of the 151-bp island in the *ESR1* gene: GRCh38: Chr6: 151,500,579–151,500,730

According to our analysis, the proximal promoter of the *ESR*1 gene contains a putative CpG island located between the transcription start site (+ 1) and the ATG codon (+ 235) (Fig. 1A, B). In order to determine its methylation status, we designed a primers pair that allowed us to amplify a single 151-bp amplicon (Fig. 1C) using genomic DNA from samples of patients diagnosed with fibroadenoma. Furthermore, a band of approximately 400 bp was detected in negative methylated controls, which is attributed to the non-specificity of the primers used in our study.

**Fig. 2:**
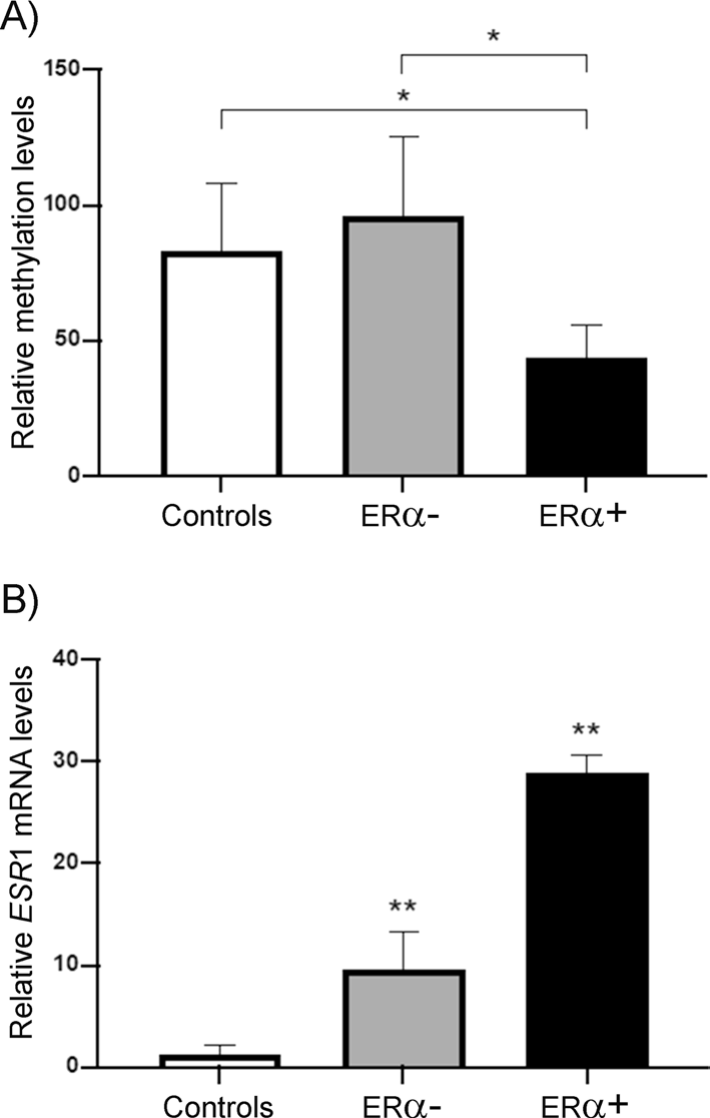
Methylation levels at the *ESR1* CpG island among Mexican BC patients. **A** The methylation levels were significantly lower in ERα+ than in controls or ERα− samples (*p < 0.05). **B** *ESR*1 transcript levels. Relative *ESR*1 mRNA amount was determined by qRT-PCR and normalized with *18S* RNA gene expression. Data represented the mean ± SD of three independent experiments (**p < 0.05)

We compared *ESR*1 methylation levels in controls with BC samples classified into ERα+ or ERα− (Fig. 2A). Controls had no difference with ERα− samples. In contrast, methylation in ERα+ samples was half that controls or ERα− samples (p < 0.05) (Fig. 2A). As expected, *ESR*1 mRNA expression was inversely proportional to the methylation status (Fig. 2B). Controls and ERα− samples with the higher methylation levels exposed a minimal amount of *ESR*1 mRNA, while the higher mRNA quantity corresponded to ERα+ samples. Interestingly, in BC samples, we found a difference between the ERα+ and ERα− samples in terms of *ESR*1 mRNA expression levels, the quantity of *ESR*1 mRNA in ERα+ samples was threefold higher than in ERα− samples (p < 0.05) (Fig. 2B). Representative immunohistochemical images of control patients and case ERα– and ERα+ patients are shown in Additional file 1: Fig. S1.

**Fig. 3:**
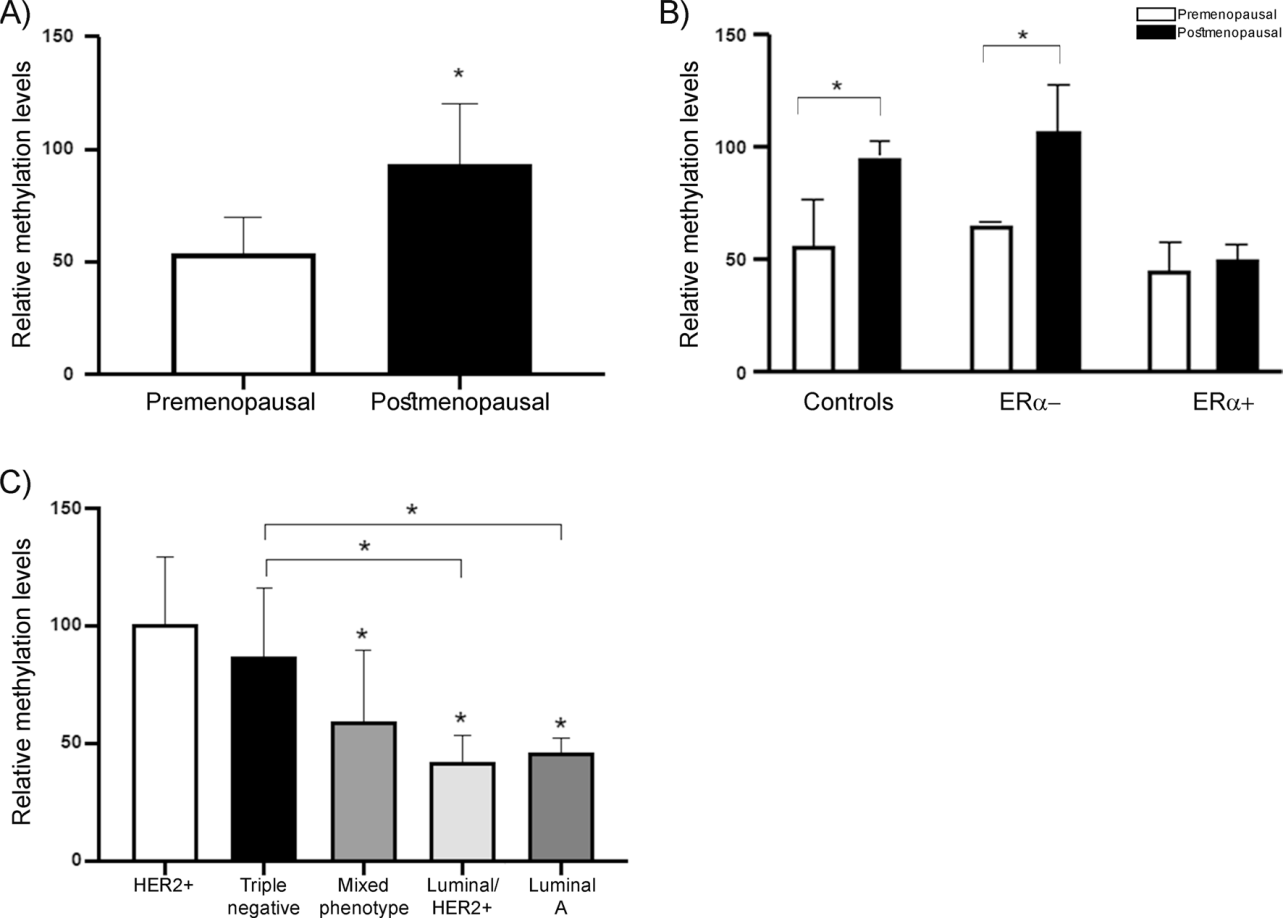
Methylation levels at the *ESR*1 CpG island between patients’ subgroups. **A** Premenopausal and postmenopausal subgroups. Methylation levels were significantly higher in postmenopausal than in premenopausal samples (*p < 0.05). **B** Methylation level for pre and postmenopausal women. Controls and BC subgroups were analyzed separately by methylation state, showing that methylation levels of controls and ERα− samples belonging to the postmenopausal subgroup (black), were significantly higher than in premenopausal (white) (*p < 0.05). **C** Tumor phenotype subgroups. Methylation levels at *ESR*1 were higher in HER2+ and triple-negative phenotypes and lowered in luminal A and luminal/HER2+ phenotypes. HER2+ samples were significantly different from luminal A, luminal/HER2+, and mixed phenotypes (*p < 0.05), while triple-negative phenotype was statistically different than luminal A and luminal/HER2+ phenotypes (*p < 0.05)

Moreover, we analyzed the methylation levels taking together all samples (Fig. 3A) and separately by menopausal stage and controls (Fig. 3B). According to ANOVA analysis, methylation in the postmenopausal women subgroup was twofold higher than in premenopausal women (p < 0.05) (Fig. 3A). Furthermore, the methylation levels of pre and postmenopausal women were analyzed separately (Fig. 3B). We observed significant differences in the methylation levels of the novel CpG island in controls and ERα− samples. Data showed that methylation levels in controls and ERα− samples belonging to the postmenopausal subgroup were significantly higher than in premenopausal women of those subgroups (*p < 0.05). Likewise, in tumor phenotype subgroup analyses, samples classified as HER2+ and TNBC phenotypes revealed higher relative methylation levels (Fig. 3C). The pairwise comparisons among the HER2+ and all subgroups indicated that methylation level in the HER2+ subgroup was higher than in mixed phenotype (p = 0.0237), luminal/HER2+ (p = 0.0002), and luminal A phenotypes (p = 0.0035). Additionally, methylation levels in TNBC phenotype samples were significantly higher than in luminal/HER2+ (p = 0.0054) and luminal A (p = 0.0131) phenotypes (Fig. 3C). Finally, statistical correlation tests suggested that the studied population's other clinicopathological characteristics were not associated with methylation levels at this region of the *ESR*1 gene. However, in the immunohistochemical profile analysis, the expression of HER2+ was statistically different (*P* = 0.0457) (Table 1).

## Discussion

Estrogens exert a vast range of biological effects in menopausal women, and their receptors, such as ERα, are a critical BC prognostic biomarker [26]. *ESR*1 gene has a complex regulation due to its regulatory elements, such as promoters with tissue-specificity [27].

We analyzed the promoter A of *ESR*1 to find sequences that undergo epigenetic modifications. We identified a 151-bp sequence located from + 29 to + 180, considering the transcription start site of *ESR*1 that begins from chr6:152,128,814–152,129,050 based on the genomic coordinates previously described [28]. Interestingly, the CpG island reported here is located between two different functional islands, one situated at promoter A (− 403 to − 266) and the other one located at exon 1 (+ 356 to + 549) [29].

We investigated the association of the methylation at the 151-bp CpG island with the expression of ERα. Our results suggested that methylation of this novel island depends on ER but not on the estrogen binding. As previously reported, methylation of *ESR1* in proximal regulatory regions was not associated with estradiol levels [30, 31]. Since the 151-bp island is in a proximal promoter, its degree of methylation might not be associated with estradiol levels.

Our results suggested that postmenopausal women have a higher susceptibility to BC than premenopausal women (P = 0.0016). In these patients, the methylation levels of 151-bp island were higher in comparison with premenopausal ones. Therefore, we suggested that the low methylation level of this island is associated with ER-. Furthermore, the methylation level of the identified CpG island in postmenopausal women could be linked with a predisposition to developing BC.

According to statistics, most BC cases are classified as ER-positive, and the ER-negative tumors are declining [32]. Our findings indicated that methylation in ERα+ samples are half that found in controls or ERα− samples, suggesting that the high expression of *ESR*1 mRNA might be related to the rising number of ER-positive BC cases. *ESR*1 expression in ER-positive tumors is associated with longer relapse-free survival time [33].

Gene expression is regulated through several mechanisms, such as epigenetic modifications and post-translational modifications. Furthermore, transcriptional regulation of *ESR*1 is critical in controlling ER expression [34]. Our results showed evidence that methylation at the novel CpG island might be associated with mRNA expression.

Methylation levels in BC Mexican patients classified as ERα− were twofold higher than in ERα+ samples, while *ESR*1 mRNA in ERα+ tumors were threefold higher than in ERα− samples. Interestingly, 20–25% of ER-negative BC samples express detectable levels of *ESR*1 mRNA [35]. Here, we reported a similar result; we found a considerable amount of *ESR*1 mRNA in ERα− samples. According to previous reports, the ER expression might be regulated at different levels in these tumors, such as posttranscriptional or post-translational mechanisms [35–37]. Our findings suggested that in ERα− tumors, methylations levels in the 151-bp island are higher compared to ERα+. Inversely, the *ESR1* mRNA expression is lower in ERα− compared with ERα+. These data suggested that *ESR1* gene expression in BC is a complex process regulated at several levels that might include methylation at 151-bp island in the proximal promoter region and its chromatin environment [34]. In addition to the high methylation status of *ESR*1 in samples classified as ERα− and controls, we observed a minimal amount of *ESR*1 mRNA in those. Then, approximately 3% of BC patients contain amplifications of the *ESR*1 gene [38, 39], suggesting the importance of regulation of *ESR*1 expression. Methylation of ER genes revealed a decrease in the levels of ERs proteins [40]. Since ERα protein expression diminished with increases in the methylation at *ESR*1 [41], we might suggest that decreased levels of mRNA *ESR1* in ERα− samples and controls were due to methylation of the 151-bp CpG island and chromatin components involved in the basal activity of the *ESR1* gene.

Interestingly, we found an association between ESR1 methylation at 151-bp island and postmenopausal BC patients. This CpG island is located in the middle of two neighboring CpG islands, which showed a high methylation level in obese postmenopausal healthy women [29]. There might be an association between methylation at the region close to the transcription start site in the *ESR*1 gene and postmenopausal stage regardless of the BC's presence. Since methylation might occur as an early initiation event or even BC development, quantifying methylation levels at 151-bp island might be a crucial tool for better stratification of tumor subtype in the Mexican population from the country's center. It is noteworthy to mention that Mexico shows genetic differences mainly due to the Amerindian and European contributions; however, in the center of the country, the principal origin of the patients included in this study is *mestizo* [42].

For the reasons stated, we reported an association between the methylation at 151-bp island of the *ESR*1 gene and immunohistochemical tumor characterization. Previous reports suggested that ER-positive tumors, such as luminal A, are linked with long-term survival, whereas ER-negative subtypes such as TNBC and HER2+ had poor prognoses [43].

Remarkably, luminal A and luminal A/HER2+ subtypes presented diminished *ESR*1 methylation levels, whereas TNBC and HER2+ tumors had increased levels, suggesting a possible relationship between high methylation levels at 151-bp island in the *ESR*1 gene and poor prognosis. Therefore, methylation levels at the novel CpG island might be related to tumor subtype resulting in an additional tool for better stratification of BC.

Although BC subtypes are commonly determined by molecular expression and hormone indicators (among others), their stratification is still challenging due to the heterogenicity of BC at histological and molecular levels. In order to differentiate ER+ from luminal A or B tumors, a gene expression profiling (GEP) of about 500 genes is used [44].

Methylation signatures in BC subtypes have been used for tumor stratification [45–47]. Since methylation levels at the 151-bp CpG island in HER2+ and TNBC are higher in comparison with luminal ones, the quantification of methylation status at this novel CpG island might be used as an additional screening assay to better BC stratification in Mexican women.

Until now, 87 distinct DNA methylation biomarkers had been reported; among them, 68 markers were analyzed once in a single population [48], exposing the need for biomarkers for a specific community as Mexican women. Some reports indicate that methylation at *ESR*1 has no statistically significant correlation with BC outcome [15, 41, 49–52]. However, our findings suggested that methylation levels in the 151-bp island from that gene might be implemented to assist BC stratification. Nowadays, one of the challenges facing the use of DNA methylation as a potential biomarker is to define the precise genomic location, the effect of DNA methylation on gene expression and its ability to change throughout the patient's life [53]. Therefore, our evidences could be the first step for an epigenetic biomarker proposal, which has been a growing field in clinical research.

## Conclusion

DNA methylation biomarkers together with histochemical characterization are helpful tools for BC prognosis. Since high methylation levels at the 151-bp CpG island into the *ESR*1 gene are associated with the postmenopausal stage and poor prognosis in BC Mexican patients, methylation at this island might be a potential prognostic biomarker in the Mexican population. In BC, molecular biomarkers predict the effectiveness of therapies and prognosis. Until now, the biomarkers used for this purpose include ER, PR, and HER2, which are tested by immunohistochemical analysis. However, these results might be subject to interpretation.

Our results suggest that the use of molecular tools such as the methylation status of 151-bp on the proximal promoter of ESR1 gene could contribute to a better understanding of the BC stratification, allowing to go one step further in the search for useful prognosis biomarkers for breast cancer patients.

## Supplementary Information

**Additional file 1: Fig. S1.** Expression of ERα in the mammary gland determined by immunodetection. Representative images correspond to control patients (**a**), case ERα− patients (**b**–**d**), and case ERα+ patients (**e**–**g**). Histological analysis was performed on paraffin-embedded mammary gland and imaged at 40×. Scale bar = 50 µm.

## Data Availability

Contact the corresponding author to have access to the data that served to support the results found in this study.

## Abbreviations

BC: Breast cancer
CpG: DNA methylation of cytosine-guanine dinucleotides
ERα: Estrogen receptor-alpha
ESR1: Estrogen receptor 1
TIMP3: TIMP metallopeptidase inhibitor 3
RASSF1A: Ras association domain family 1 isoform A
RARβ: Retinoic acid receptor β
DNMT1: DNA methyltransferases 1
DNMT3A: DNA methyltransferases 3A
TNBC: Triple-negative breast cancer
PR: Progesterone receptor
chr6: Chromosome 6
ER+: Estrogen receptor-positive
ER−: Estrogen receptor-negative
