# Supplementary figures and images for: The high methylation level of a novel 151-bp CpG island in the ESR1 gene promoter is associated with a poor breast cancer prognosis
Source: Cancer Cell Int. 2021 Dec 4;21:649. doi: 10.1186/s12935-021-02343-7 (PMC8645138; doi:10.1186/s12935-021-02343-7)

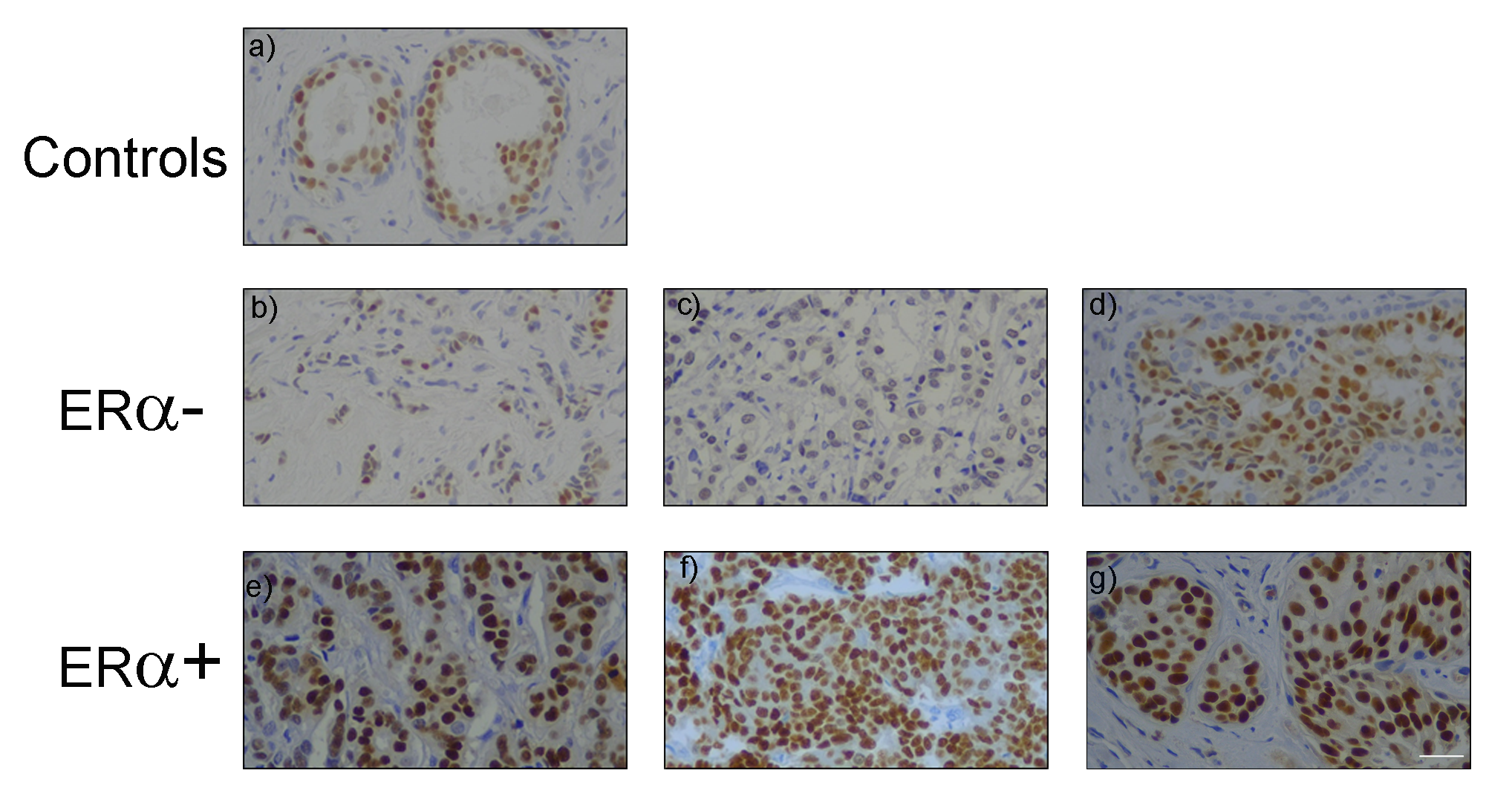

Supplement: Supplementary file 1 — Additional file 1: Fig. S1. Expression of ERα in the mammary gland determined by immunodetection. Representative images correspond to control patients (a), case ERα− patients (b–d), and case ERα+ patients (e–g). Histological analysis was performed on paraffin-embedded mammary gland and imaged at 40×. Scale bar = 50 µm. [file 12935_2021_2343_MOESM1_ESM.tif]
